# Rev-erb-α regulates atrophy-related genes to control skeletal muscle mass

**DOI:** 10.1038/s41598-017-14596-2

**Published:** 2017-10-30

**Authors:** Alicia Mayeuf-Louchart, Quentin Thorel, Stéphane Delhaye, Justine Beauchamp, Christian Duhem, Anne Danckaert, Steve Lancel, Benoit Pourcet, Estelle Woldt, Alexis Boulinguiez, Lise Ferri, Mathilde Zecchin, Bart Staels, Yasmine Sebti, Hélène Duez

**Affiliations:** 1 Univ. Lille, Inserm, CHU Lille, Institut Pasteur de Lille, U1011 - EGID, F-59000 Lille, France; 2 Institut Pasteur, Imagopole - CITech, Paris, France

## Abstract

The nuclear receptor Rev-erb-α modulates hepatic lipid and glucose metabolism, adipogenesis and thermogenesis. We have previously demonstrated that Rev-erb-α is also an important regulator of skeletal muscle mitochondrial biogenesis and function, and autophagy. As such, Rev-erb-α over-expression in skeletal muscle or its pharmacological activation improved mitochondrial respiration and enhanced exercise capacity. Here, in gain- and loss-of function studies, we show that Rev-erb-α also controls muscle mass. Rev-erb-α-deficiency in skeletal muscle leads to increased expression of the atrophy-related genes (atrogenes), associated with reduced muscle mass and decreased fiber size. By contrast, *in vivo* and *in vitro* Rev-erb-α over-expression results in reduced atrogenes expression and increased fiber size. Finally, Rev-erb-α pharmacological activation blocks dexamethasone-induced upregulation of atrogenes and muscle atrophy. This study identifies Rev-erb-α as a promising pharmacological target to preserve muscle mass.

## Introduction

Skeletal muscle is the most abundant tissue in the body, accounting for 30 to 40% of total body mass. Besides its role in locomotion and posture, skeletal muscle controls whole body energy metabolism by ensuring blood glucose control and is also the major source of amino acids (AAs) that can be used by various tissues during prolonged periods of fasting. Skeletal muscle is highly plastic and muscle mass, fiber type and size can undergo major remodeling to meet developmental and environmental challenges^1^. Muscle mass increases during development and in the adult in response to mechanical overload or hormonal stimulation, whereas muscle wasting is a common condition in diverse pathologies including obesity/type 2 diabetes, cancers, myopathies, chronic inflammatory disorders and is also a hallmark of aging, undernutrition or inactivity^2,3^. Chronic glucocorticoid therapies for the treatment of inflammatory or auto-immune disorders are also characterized by a rapid muscle atrophy. Loss of muscle mass results in reduced ability to sustain contraction, weakness of peripheral and respiratory muscles that compromises mobility and negatively affects quality of life. Muscle wasting also results in poor prognosis in diverse diseases, and ultimately has major clinical implications^3^. Therefore, understanding the signaling pathways that control muscle mass is of special interest for the development of novel therapies to preserve muscle mass.

Skeletal muscle mass reflects the delicate balance between protein synthesis and degradation. Protein synthesis is essentially controlled by the insulin-like growth factor 1– phosphoinositide-3-kinase–Akt/protein kinase B–mammalian target of rapamycin (IGF1-PI3K-Akt-mTOR) signaling pathway in response to a variety of stimuli such as IGF1, insulin, AAs derived from protein degradation, β2-adrenergic receptor agonists or androgens^1^. Activation of PI3K induces Akt phosphorylation which, in turn, inhibits the protein complex TSC1 (Tuberous Sclerosis Complex 1)-TSC2 thus activating the mTOR pathway through the two main downstream effectors of mTORC1: S6K (p70S6 kinase) and 4E-BP1 (eIF-4E-binding protein)^4^. pAkt also negatively regulates protein degradation by phosphorylating the Forkhead box O (FoxO) transcription factors and by promoting their exclusion from the nucleus^1^.

Protein degradation occurs via two proteolytic pathways that are tightly regulated by signaling pathways and transcription factors: the ubiquitin-proteasomal system, which predominantly breakdowns myofibrillar proteins, and the autophagic-lysosomal machinery, which ensures the maintenance of cellular homeostasis and the physiological turnover of damaged/aged cellular organelles^1,3^. Upon atrophy signals, FoxOs induce the transcription of the two E3 ubiquitin ligases MuRF1 (Muscle-Specific RING Finger Protein 1) and Atrogin-1 (MAFbx) to promote proteasomal protein degradation^5,6^. Mouse models invalidated for *atrogin-1* or *Murf1* are resistant to starvation and glucocorticoid (GC)-induced atrophy, respectively^7–10^. The FoxO family is composed of three isoforms FoxO1, FoxO3a and FoxO4 and *in vivo* upregulation of FoxO1 and FoxO3 is sufficient to promote muscle atrophy^6,11–13^. In addition, FoxO activation also induces autophagy^14,11^, a process tightly regulated at the gene, protein and flux levels and involving several autophagy-related genes (atg) as well as proteins such as Parkin and Bnip3 which contribute to mitochondrial remodeling^15^.

Circulating levels of GC are increased in diseases associated with muscle atrophy like sepsis or cachexia, and promote muscle mass loss^16,17^. Similarly, administration of synthetic GCs, such as dexamethasone used for the treatment of chronic inflammatory and immune diseases, results in muscle atrophy^18^. GCs promote both protein proteasomal degradation and autophagy-mediated proteolysis^7–9^. In addition, GCs exert anti-anabolic actions by up-regulating Regulated in Development and DNA damage responses 1 (Redd1, also known as ddit4) and Kruppel Like Factor 15 (KLF15), which directly repress mTOR signalling^9,19,20^. Beside, KLF15 acts on mTOR activity through the up-regulation of Branched Chain Amino Acid Transaminase 2 (BCAT2), a mitochondrial enzyme that degrades branched chain AAs. KLF15 also enhances *Murf1* and *atrogin-1* gene expression^9^.

The nuclear receptor Rev-erb-α acts as a transcriptional repressor by recruiting co-repressors and binding to specific DNA regions of its target genes^21,22^ or by recruiting co-repressors to sites to which it is tethered by tissue-specific transcription factors^23^. We and others have shed light on the role of Rev-erb-α in the circadian control of hepatic lipid, bile acid and glucose metabolism^21,22,24–27^, white adipocyte *in vitro* differentiation^28,29^, as well as brown adipocyte thermogenesis^30^. We have recently shown that Rev-erb-α controls skeletal muscle autophagy and mitochondrial biogenesis. Moreover, pharmacological activation of Rev-erb-α *in vivo* increases skeletal muscle mitochondrial respiration and exercise capacity, identifying Rev-erb-α as a target to improve muscle function^31^. Whether Rev-erb-α controls muscle mass has not been investigated yet.

Here, we report that *Rev-erbα* ^−/−^ mice display significantly reduced skeletal muscle mass and decreased fiber cross section area. This is associated with an increased expression of atrophy-related genes in skeletal muscle from *Rev-erbα* ^−/−^ mice, while AAV-mediated skeletal muscle-specific-Rev-erb-α overexpression confers an opposite phenotype. This effect on gene expression and fiber size was recapitulated in *in vitro* models of Rev-erb-α gain- and loss-of-function in myogenic cells. In addition, Rev-erb-α over-expression and/or pharmacological activation partially or totally blunted the induction of atrophy genes by dexamethasone in a mouse myoblast cell line as well as muscle mass loss *in vivo*. Together these data point to a new role for Rev-erb-α in the control of skeletal muscle mass and suggest that pharmacological activation of Rev-erb-α may constitute a promising approach to limit muscle wasting and improve the quality of life and survival of patients.

## Results

### *Rev-erbα*^−/−^ mice display reduced muscle mass associated with smaller muscle fibers

**Figure 1:**
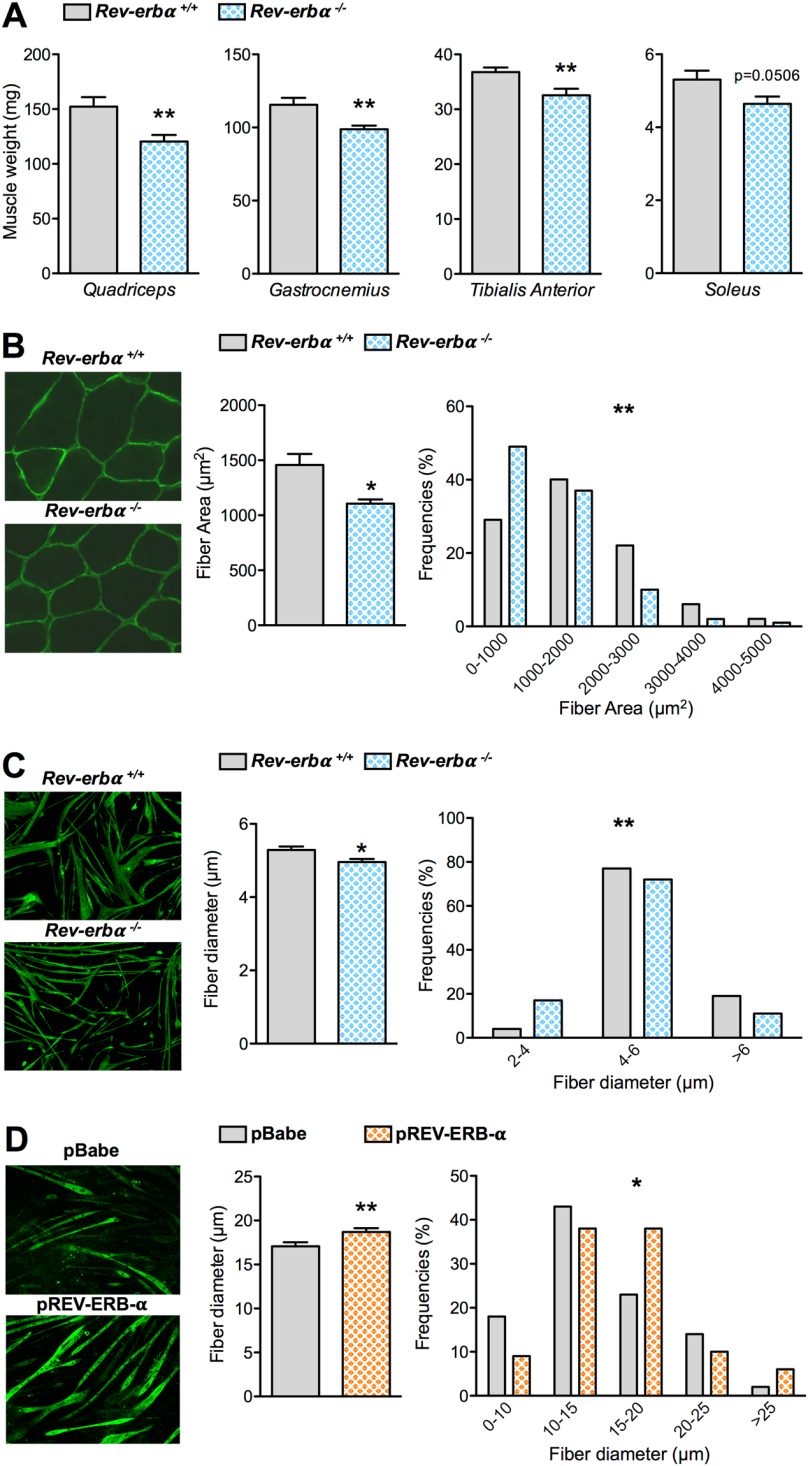
Rev-erb-α controls skeletal muscle mass. (**A**) Quadriceps, gastrocnemius, tibialis anterior and soleus muscle mass from *Rev-erbα* ^+/+^ and *Rev-erbα* ^−/−^ mice (n = 13 and 11, respectively). (**B**) Representative immunostaining of laminin on skeletal muscle sections (left) and mean fiber cross-sectional area and fiber area distribution of tibialis anterior muscle from *Rev-erbα* ^+/+^ and *Rev-erbα* ^−/−^ mice (>13 000 fibers per mouse, n = 4 mice per genotype) (right). (**C**) Representative immunostaining of myosin Heavy Chain (left) and mean fiber diameter and fiber diameter distribution of fibers differentiated from myogenic precursors isolated from *Rev-erbα* ^+/+^ and *Rev-erbα* ^−/−^ mice (3 measures per fiber, n = 120 fibers per genotype) (right). (**D**) Representative immunostaining of Myosin Heavy Chain (left) and mean fiber diameter and fiber diameter distribution of differentiated C2C12 cells over-expressing Rev-erbα (*p*REV-ERB-α) and control cells (pBabe) (3 measures per fiber, n = 120 fibers per group) (right). Results are expressed as means ± sem; *p < 0.05, **p < 0.01 by *t*-test (**A**), Mann-Whitney test (**B**–**D**) or chi-square test (**B**–**D**) to compare, respectively, the mean or the frequency distribution between groups.

Here, we explored whether Rev-erb-α plays a role in the control of skeletal muscle mass or fiber size. Different hindlimb skeletal muscles were harvested from the *Rev-erbα* ^−/−^ mice and their *Rev-erbα* ^+/+^ littermates and weighed. Quadriceps, gastrocnemius and tibialis anterior (TA) mass was significantly lower (by −20.9%, −14.5% and −11.5%, respectively) in *Rev-erbα* ^−/−^ compared to *Rev-erbα* ^+/+^ mice (Fig. 1A), whereas body weights were similar (Figure S1). The weight of the soleus muscle, which mostly contains oxidative fibers, was also reduced in *Rev-erbα* ^−/−^ mice compared to *Rev-erbα* ^+/+^ mice (by 12.6%) although the difference did not reach statistical significance (Fig. 1A). This is in accordance with the fact that glycolytic muscles are generally more vulnerable to atrophy than oxidative muscles^19^. Interestingly, *Rev-erbα* ^+/−^ mice which have normal locomotor activity also display a tendency to reduced muscle mass compared to the wild-type mice (data not shown), thus indicating that reduced muscle mass occurs independently of any changes in the locomotor activity.

To further characterize the impact of Rev-erb-α on skeletal muscle mass, skeletal muscle fiber cross sectional areas (CSA) were quantified on TA sections using a laminin staining which surrounds muscle fibers. In line with the reduction of muscle mass, the mean fiber CSA was significantly lower in *Rev-erbα* ^−/−^ compared to *Rev-erbα* ^+/+^ mice with a shift in the fiber size distribution toward smaller fibers (Fig. 1B). These data therefore indicate that skeletal muscle is atrophied in absence of Rev-erb-α.

Next, primary myofibers were differentiated *in vitro* from myogenic precursors isolated from *Rev-erbα* ^+/+^ and *Rev-erbα* ^−/−^ mice to assess the role of Rev-erb-α exclusively in myogenic cells. As observed *in vivo*, the mean muscle fiber diameter was significantly lower in *Rev-erbα*-deficient cells, again with a shift toward smaller fibers (Fig. 1C). Next, fiber diameter was measured on *in vitro* differentiated C2C12 overexpressing Rev-erb-α (pREV-ERB–α) and control C2C12 myofibers (pBabe). Expectedly, a mirror phenotype was observed, ie the mean fiber diameter was significantly higher in Rev-erb-α overexpressing fibers compared to controls, with a decrease in smaller fiber size and an increase in larger fibers (Fig. 1D). Thus, muscle mass and fiber size changes reflect a direct, cell autonomous, impact of Rev-erb-α on fibers rather than an adaptive response to a chronic situation.

### Rev-erb-α negatively regulates catabolic and anti-anabolic pathways in skeletal muscle

**Figure 2:**
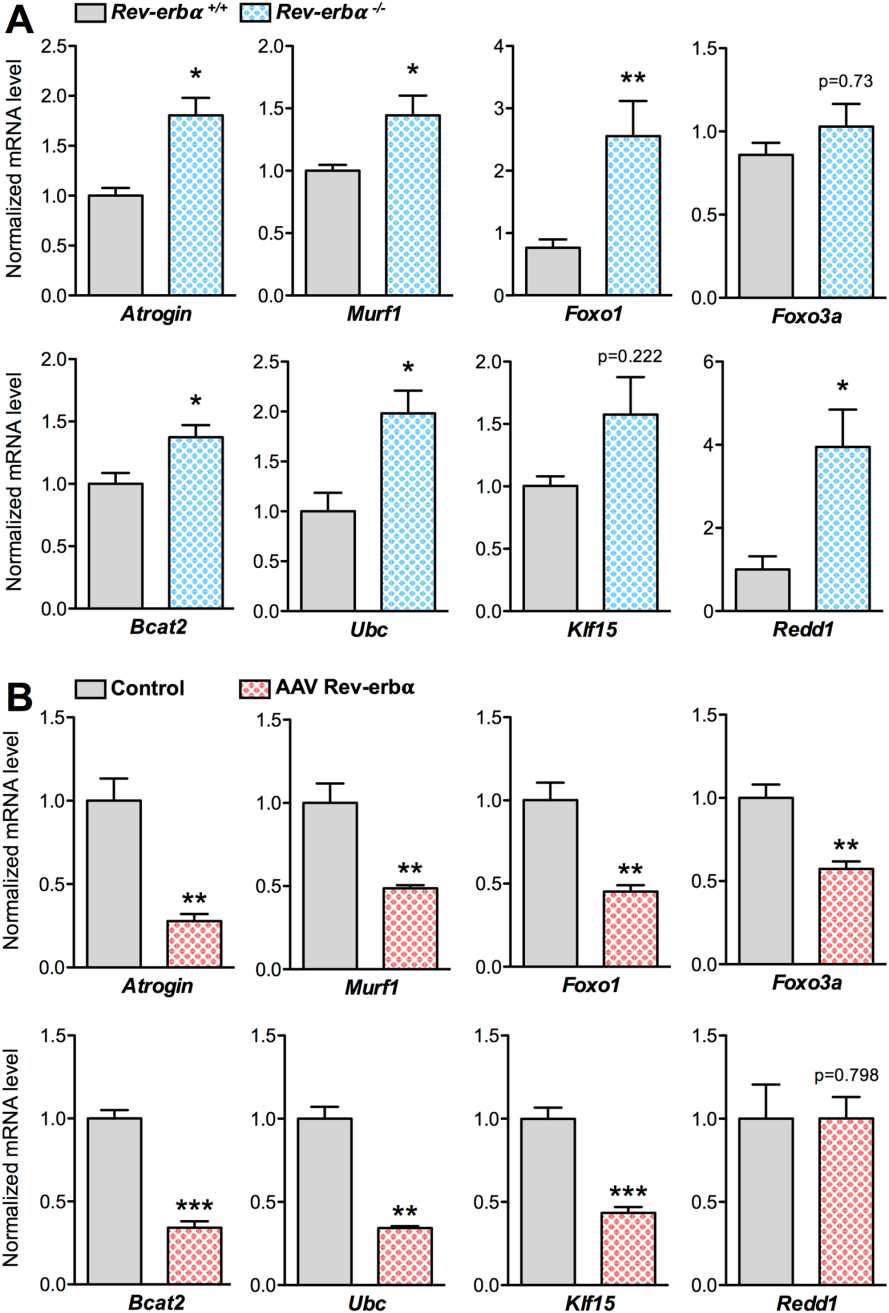
Rev-erb-α controls atrophy-related genes in skeletal muscle. (**A**) RT-qPCR analysis of atrophy-related gene expression in quadriceps muscle from *Rev-erbα* ^+/+^ and *Rev-erbα* ^−/−^ mice (*n* = 5 per genotype). (**B**) RT-qPCR analysis of atrophy-related gene expression in gastrocnemius muscle from mice intra-muscularly injected with a Rev-erb-α expressing or a control AAV vector (n = 7 per group). Results are expressed as means ± sem; *P < 0.05, **P < 0.01, ***P < 0.001 by Mann-Whitney test.

To determine whether these changes in muscle mass and fiber size are due to a dysregulation of the transcriptional programs elevated in muscle atrophy, expression analysis of the genes belonging to these processes was performed on skeletal muscles isolated from both Rev-erb–α loss- and gain-of-function mouse models. In particular, expression of genes encoding proteins involved in the ubiquitin-proteasome system was measured. Our results show that expression of the catabolic genes *ubiquitin* (*Ubc*), encoding the polyubiquitin precursor, and the two E3 ubiquitin ligases *Atrogin* and *Murf1*, as well as their transcriptional regulators *Foxo1* and *Foxo3a* is significantly increased in muscle from *Rev-erbα* ^−/−^ mice compared to their *Rev-erbα* ^+/+^ littermates (Fig. 2A). By contrast, AAV-mediated Rev-erb-α over-expression in wild-type mice (Figure S2A) led to the mirror phenotype, namely a significant downregulation of these catabolic genes (Fig. 2B, Figure S2). These results are in accordance with the increase in proteosomal activity in myofibers obtained after differentiation of myogenic precursor isolated from *Rev-erbα* ^+/+^ and *Rev-erbα* ^−/−^ mice and the mirrored decreased proteasomal activity measured in C2C12 cells overexpressing Rev-erb-α (Figure S3).

We also examined the expression of *Redd1 and Klf15*, two genes encoding negative regulators of the anabolic mTOR signaling pathway and known glucocorticoid receptor (GR) targets^9^. Expression of both genes was upregulated in skeletal muscle in absence of *Rev-erbα* (Fig. 2A), while Rev-erb-α over-expression decreased expression of these genes (Fig. 2B, Figure S2B). Accordingly, expression of *Bcat2*, a downstream *Klf15* target gene, was significantly upregulated in *Rev-erbα* ^−/−^ skeletal muscle, and down-regulated upon Rev-erb-α over-expression (Fig. 2A,B, Figure S2B). These alterations occurred in absence of any changes in the mTOR signaling pathway assessed by western blot (data not shown).

Altogether with our previous observation that Rev-erb-α down-regulates skeletal muscle autophagy^31^, these findings indicate that Rev-erb-α represses catabolic and anti-anabolic pathways in skeletal muscle, and that its absence leads to skeletal muscle atrophy through the activation of these pathways.

### Rev-erb-α over-expression and pharmacological activation counteracts atrophy induced by dexamethasone

**Figure 3:**
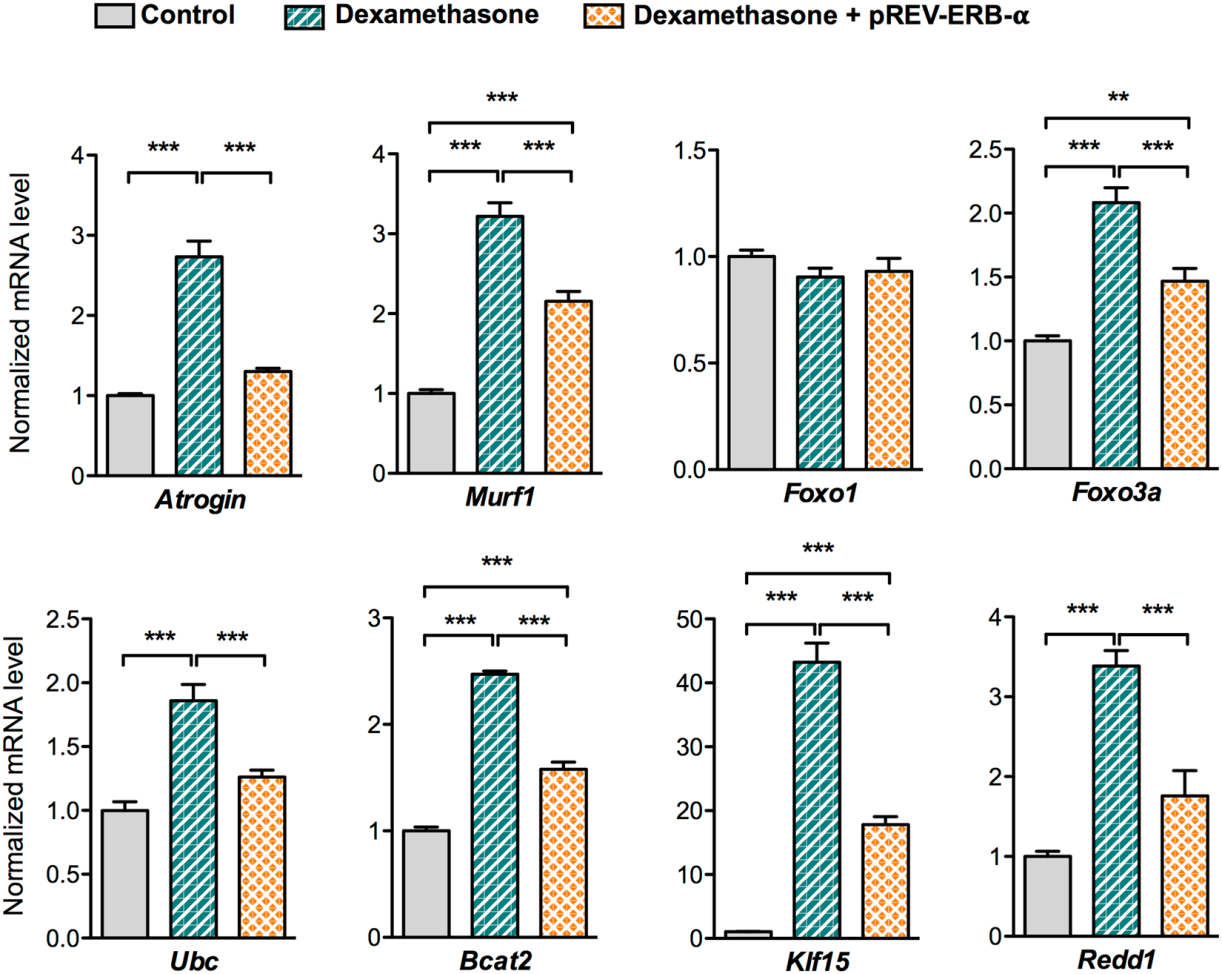
Rev-erb-α over-expression counteracts dexamethasone-mediated induction of atrophy-related genes. RT-qPCR analysis of atrophy-related gene expression in differentiated C2C12 cells infected with Rev-erb-α or control retrovirus and treated with dexamethasone (1 µM) or vehicle for 48hrs (n = 6 per condition). Results are expressed as means ± sem; **P < 0.01, ***P < 0.001 by One-way ANOVA with Bonferroni post-hoc analysis.

**Figure 4:**
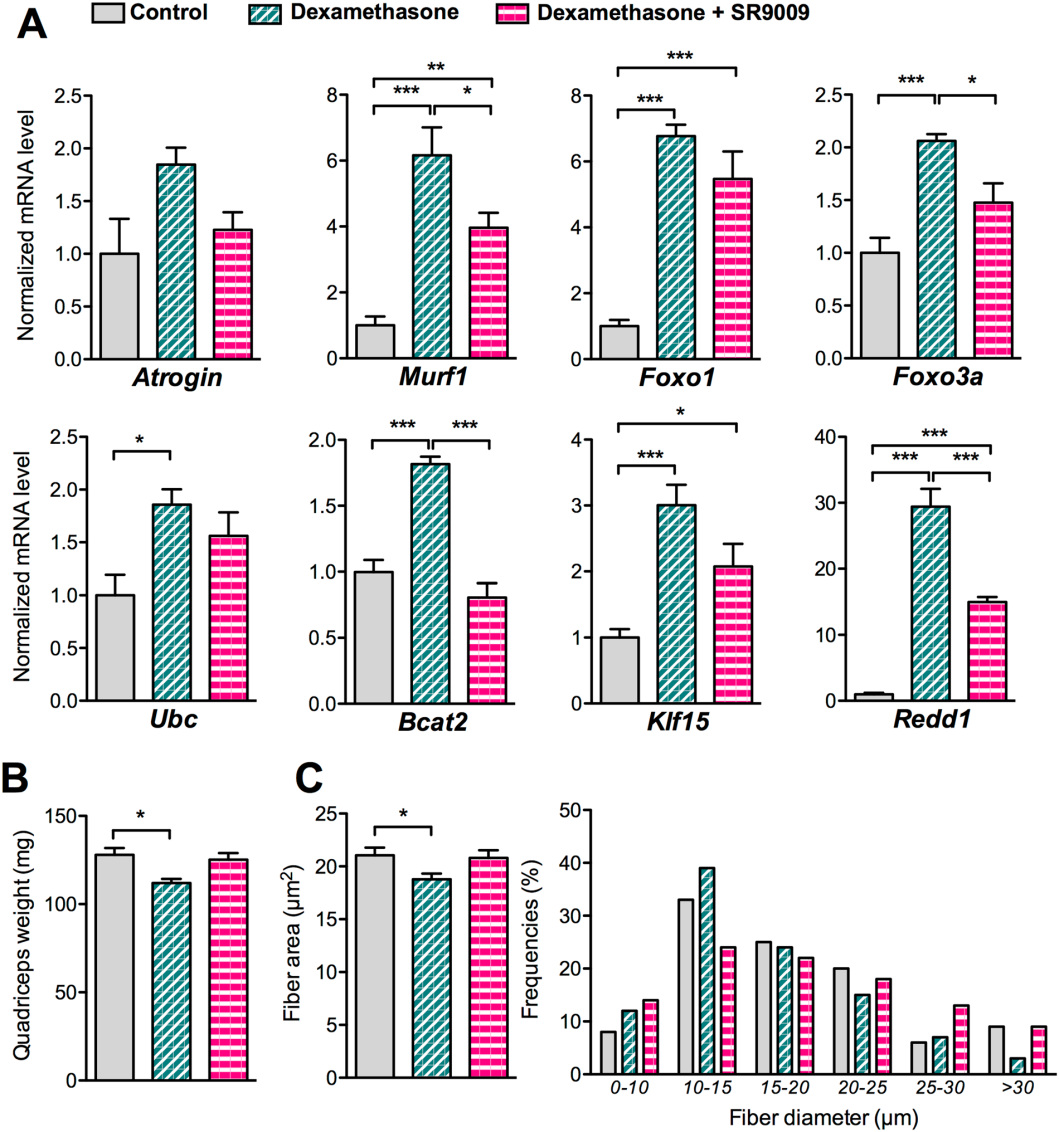
Pharmacological Rev-erb-α activation preserves muscle mass upon dexamethasone treatment. (**A**) RT-qPCR analysis of atrophy-related gene expression in quadriceps muscle (n = 5 per group) and (**B**) quadriceps muscle mass (n = 4 per group) from mice receiving or not 10 mg/kg/day of dexamethasone and co-treated with SR9009 (100 mpk) twice daily or vehicle for 3 days. (**C**) Mean fiber diameter and fiber size distribution of differentiated C2C12 cells treated or not with dexamethasone (20 µM) and co-treated with SR9009 (10 µM) or vehicle for 3 days (3 measures per fiber, n = 120 fibers per group). Results are expressed as means ± sem; *p < 0.05, **p < 0.01, ***p < 0.001 by One-way ANOVA with Bonferroni post-hoc analysis or chi-square test to compare respectively the mean or the frequency distribution between groups.

Chronic dexamethasone treatment induces skeletal muscle fibers atrophy through the activation of different genes found to be regulated by Rev-erb-α. Therefore, we tested whether Rev-erb-α can counteract dexamethasone-induced muscle wasting. To this aim, Rev-erb-α over-expressing and control C2C12 myogenic cells were differentiated and then treated during 48 hrs with dexamethasone. Rev-erb-α overexpression partially or totally blunted dexamethasone-mediated induction of the catabolic (*Atrogin*, *Murf1*, *Foxo1* and *Foxo3a*) and anti-anabolic (*Klf15*, *Redd1* and *Bcat2)* genes (Fig. 3). Rev-erb-α is a ligand-activated nuclear receptor, and we have previously demonstrated that its pharmacological activation with the SR9009 compound has beneficial effects on skeletal muscle endurance training^31^. Therefore, we next assessed whether Rev-erb-α activation may preserve muscle mass upon glucocorticoid administration *in vivo*. To this aim, wild-type mice were treated with dexamethasone alone or with the Rev-erb agonist SR9009 for 3 days. As expected, dexamethasone treatment potently induced the expression of the catabolic genes (*Atrogin*, *Murf1*, *Ubc*, *Foxo1* and *Foxo3a*) as well as the well-known *Klf15*, *Redd 1* and *Bcat2* GR target genes (Fig. 4A). Interestingly, co-treatment of the mice with the SR9009 Rev-erb-α ligand partially or totally blunted the induction of these genes by dexamethasone (Fig. 4A), resulting in muscle mass preservation (Fig. 4B). Quantification of muscle fiber diameter was performed upon 3 days treatment with dexamethasone on fully differentiated cells. Fiber diameter was strongly reduced upon dexamethasone treatment, and SR9009 co-treatment fully prevented this effect (Fig. 4C). Altogether, these data indicate that Rev-erb-α overexpression or its pharmacological activation abolishes dexamethasone atrophic effects *in vitro* and *in vivo*.

### Rev-erb-α controls FoxOs nuclear translocation and directly targets atrophy-related genes

**Figure 5:**
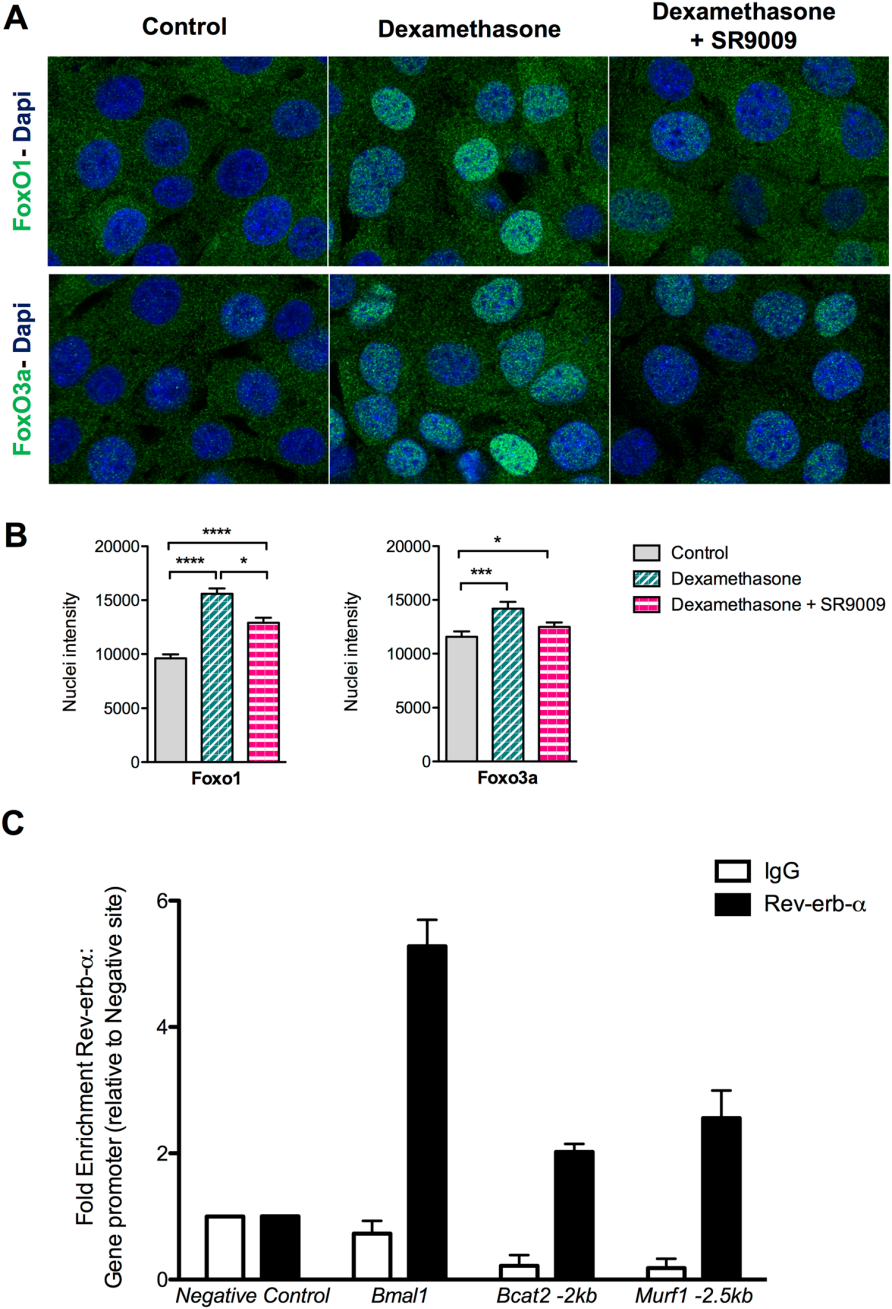
Rev-erb-α modulates FoxO nuclear translocation and directly regulates atrophy-related genes through a direct binding to their regulatory sequences. (**A**) Representative immunostaining of FoxO1 (upper panels) and FoxO3a (lower panels) in green and Dapi nuclear staining in blue of C2C12 myogenic cells treated or not with dexamethasone (1 µM) and co-treated with SR9009 (10 µM). (**B**) Nuclear intensity quantification of FoxO1 and FoxO3a (n > 100 cells per condition). Results are expressed as means ± s.e.m; *p < 0.05, ***p < 0.001, ****p<0.0001 by One-way ANOVA with Bonferroni post-hoc analysis. (**C**) Rev-erb-α binding to regulatory regions of the indicated atrophy-related genes measured by ChIP-qPCR (n ≥ 2 independent experiments). Data are expressed as means ± s.e.m.

Dexamethasone treatment promotes nuclear translocation of FoxO1 and FoxO3a. Once in the nucleus, they activate the expression of their target genes such as *Atrogin* or *Murf1*. To determine if pharmacological activation of Rev-erb-α with the SR9009 is able to modulate FoxOs cellular localization, C2C12 cells were treated with dexamethasone and co-treated or not with SR9009 and the intensity of FoxO1 and FoxO3a signal was analysed by immunofluorescence and quantified. Results show that the nuclear intensity of both FoxO1 and FoxO3a is significantly increased upon dexamethasone treatment, whereas SR9009 co-treatment blunted dexamethasone-induced FoxO1 and FoxO3a nuclear translocation **(**Fig. 5A,B).

Beside its action through FoxO signaling, Rev-erb-α may directly repress its target genes by binding specific DNA sequences in their regulatory regions. Results from ChIP experiments performed on C2C12 myogenic cells show that Rev-erb-α occupies a site located at −2 kb from the transcription star site (TSS) within the promoter sequence of *Bcat2* gene and a site located at −2.5 kb from the TSS in *Murf1* gene regulatory sequences (Fig. 5C).

In conclusion, these data show that Rev-erb-α counteracts atrophy through the modulation of FoxOs translocation, thus preventing their transcriptional activation and by a direct binding to atrophy-related genes.

## Discussion

Skeletal muscle serves several important functions in the body, from locomotion and posture to metabolic homeostasis. Muscle loss is observed in and aggravates diverse physiological (such as fasting or aging) and pathological situations (such as cancer, sepsis, inflammatory conditions), and is associated with a poor quality of life and prognosis^3^. Thus, preservation of muscle mass may have major clinical benefits.

In this study, we addressed the role of the drugable nuclear receptor Rev-erb-α in the control of muscle mass. We demonstrated that Rev-erb-α modulates muscle mass and fiber size *in vivo* and *in vitro* by repressing the expression of genes of the ubiquitin-proteasomal degradation pathway such as Ubc, the atrogin-1 and MuRF1E3 ubiquitin ligases, as well as the upstream FoxO1 and FoxO3a regulators. Our data support an intrinsic role for Rev-erb-α in the control of muscle mass. Indeed, *in vitro* observation that muscle fibers differentiated from *Rev-erbα* ^−/−^ primary myogenic progenitors display similar alterations (decreased fiber size and upregulated expression of atrophy genes), whereas Rev-erb-α over-expression in C2C12 myoblasts led to the opposite phenotype, demonstrate a cell autonomous action of Rev-erb-α in regulating muscle atrophy.

Atrogin-1 and MuRF1 mediate myofibrillar protein degradation, and nutrient-deprivation–induced and dexamethasone-induced muscle atrophy, respectively^5–7,10,32^. These two genes are regulated by FoxO transcription factors whose upregulation *in vivo* promotes muscle atrophy^6,12,13^. Indeed, the expression of a constitutively active FoxO3 mutant up-regulates *Atrogin-1* transcription and induces skeletal muscle atrophy, whereas a dominant negative FoxO3 prevents fasting-induced *Atrogin-1* expression and dexamethasone-induced atrophy in myotubes^6^. In line, *FoxO1*,*3*,*4* deletion prevented muscle loss and weakness upon fasting^11^. In our study, we show that Rev-erb-α represses these genes and controls FoxO nuclear translocation, thereby controlling muscle mass. FoxOs also control the lysosome-autophagy pathway that, beside the ubiquitin-proteasomal system, plays a critical role in skeletal muscle atrophy^33^. FoxOs mediate activation of some autophagy genes and proteins such as Bnip3, LC-3, and cathepsin L, and fasting failed to induce autophagosome formation in *FoxO*-deficient mice^11^. Interestingly, we have already demonstrated that *Rev-erbα*-deficiency in skeletal muscle increased expression of several autophagy genes such as *Bnip3*, *Pink* and *Parkin*, and increased autophagy flux as shown by increased LC3-II/LC3-I ratio^31^. This was also observed in *Rev-erbα*-deficient myoblasts, whereas Rev-erb-α over-expression led to a strong repression of autophagy genes. Besides being recruited to the promoter of several autophagy genes (*Bnip3* and *Cathepsin L*, among others)^31^, we show here that Rev-erb-α may also regulate this pathway in an indirect manner through the repression of FoxOs. Thus, our previous study and the present report demonstrate that Rev-erb-α plays a prominent role in the atrophy program by regulating the ubiquitin-proteasome and autophagy pathways in a coordinated manner.

Other transcription regulators have been shown to modulate muscle mass, amongst which the cofactor PGC1-α. *PGC1α* expression is decreased in muscle atrophy and its over-expression inhibits FoxO3 signaling, autophagy and the ubiquitin-proteasome degradation pathways without affecting protein synthesis^34^. In addition, the expression of gene encoding oxidative phosphorylation is reduced in atrophying muscle suggesting that mitochondrial dysfunction can negatively impact skeletal muscle mass maintenance by triggering catabolic pathways^35^. It is noteworthy that Rev-erb-α positively controls PGC1α expression and activity, as well as mitochondrial biogenesis and function in skeletal muscle^31^, which may contribute to the changes in muscle mass observed in the Rev-erb-α gain- and loss-of function models.

Endogenous GCs induce a moderate and reversible skeletal muscle protein catabolism in response to infection, whereas chronic GC treatment elicits muscle atrophy by increasing pathways leading to protein degradation and by antagonizing the action of anabolic factors in an exacerbated manner. In skeletal muscle, GR inhibits PI3K/Akt signaling, resulting in increased FoxOs signaling^6,36^. In addition, GR antagonizes the anabolic pathway by inducing *Redd1* expression, which leads to mTORC1 inhibition^37^, and *Klf15*, which increases the expression of the branched AAs-degrading enzyme BCAT2, thereby strengthening the inhibition of mTOR activity in an Akt-independent manner^9,38^. KLF15 also represses *Murf1* and *Atrogin-1* gene expression either directly for *Murf1* or through FoxO induction for *Atrogin-1* ^9^. Corticosterone levels are not different in Rev-erbα-deficient mice^39,40^. However, *Rev-erbα* gene expression is down-regulated in skeletal muscle upon dexamethasone treatment (Figure S4). We and others have already evidenced a regulation of *Rev-erbα* by GR and the cross-talk between these two nuclear receptors in the liver^40,41^, and it may be hypothesized that *Rev-erbα* down-regulation, or gene ablation, is permissive to GR action on atrophy genes. This is in line with the decreased muscle mass in the *Rev-erbα*-deficient mice in basal conditions and with the repression of *FoxOs* (and both proteasomal and autophagy-related genes), and of *Redd1*, *Klf15* and *Bcat2* by Rev-erb-α. In agreement with this concept, we demonstrate that Rev-erb-α pharmacological activation is able to counteract dexamethasone-mediated induction of these genes, thereby preserving muscle mass. Our study identifies Rev-erb-α as a new actor in the complex network that controls muscle atrophy and a promising target to preserve muscle mass beside its beneficial effect on exercise capacity.

## Methods

### Animals

7–12 week-old *Rev-erbα*-deficient mice (*Rev-erbα* ^−/−^) and their wild-type (*Rev-erbα* ^+/+^) littermates were studied. Mice were euthanized by cervical dislocation. Skeletal muscles (Gastrocnemius, Quadriceps, Tibialis Anterior (TA) and Soleus) were weighed and snap-frozen.

To induce skeletal muscle atrophy, 7–8 weeks old wild-type mice received an intraperitoneal injection of dexamethasone at 10 mg/kg/day or vehicle (ethanol) for 3 consecutive days at 9:00 am. Each day, half of the dexamethasone-treated mice also received two injections of the SR9009 Rev-erb agonist at 100 mg/kg at 17:30 pm the previous day and 30 min prior to dexamethasone injection. Quadriceps muscles were collected and weighed.

To induce Rev-erb-α overexpression (Figure S2A), the *Nr1d1* coding sequence was introduced in an AAV1 vector (Penn Vector Core, University of Pennsylvania) and 3 × 10^11^ genome copies were injected intra-muscularly in the TA and gastrocnemius muscles of wild-type mice as previously described^31^. The contralateral limb was injected with control AAV. 6 weeks later, mice were sacrificed and muscles were snap-frozen and stored until mRNA extraction.

Mice were housed in a 12 h/12 h light/dark cycle and had free access to food and water. All experimental procedures were performed with the approval of the Nord-Pas-de-Calais ethics committee (CEEA75) and in compliance with French and European ethical legislations.

### Myogenic Cell Culture

TA, gastrocnemius, soleus, quadriceps, diaphragm, triceps and pectoralis muscles were collected from 7–8 weeks- old *Rev-erbα*-deficient mice (*Rev-erbα* ^−/−^) and their wild-type littermates (*Rev-erbα* ^+/+^) and digested in Dulbecco’s modified Eagle’s medium (DMEM; 4.5 g/L D Glucose; Gibco) F12 medium with Collagenase D (0.15 U/mg) and Dispase II (0.8 U/mg) during 30 minutes at 37 °C. Digested muscles were then filtered on cell strainer and myogenic precursors were isolated using negative and positive selection kits (130104268 and 130104261, Miltenyi Biotec). Primary myoblasts were resuspended in DMEM F-12 and with 20% FBS, 1% gentamycin, 1 mL Ultroser G and 5 ng/mL FGFb and seeded in 0.1% gelatin coated plates. After 3 days, differentiation was induced by the addition of F-12/DMEM (1:1) medium supplemented with 5% horse serum for 7 days.

Constitutively over-expressing Rev-erb-α (*REV-ERB-α*) and control (*pBabe*) C2C12 cell lines^31^ were cultured in DMEM medium with 1% gentamycin and 10% FBS. At 80% of confluency, myogenic differentiation was induced by addition of 2% horse serum for 3 days.

For myotube atrophy experiments, C2C12 cells were cultured as previously described^31^. After 5 days of differentiation, cells were treated with dexamethasone (20 µM)^42^ or vehicle (ethanol), and SR9009 (10 µM) as indicated for 3 days. For analysis of atrophy-related gene expression, C2C12 were treated with dexamethasone (1 µM) during 2 days with or without SR9009 (10 µM). For FoxOs nuclear translocation analysis, C2C12 were treated with dexamethasone 1 µM or vehicle with or without SR9009 (10 µM) overnight.

### Immunostaining

C2C12 cells were fixed with 4% paraformaldehyde, and permeabilized with 0.2% Triton × 100/ 50 mM NH_4_Cl in PBS. Cells were then incubated with Myosin Heavy Chain (MF-20) (1:250, DSHB AB2147781), FoxO1 (1:250, Santa Cruz 2880 S) or FoxOa3 (1:250, Santa Cruz 2497S) antibodies, followed by Alexa Fluor 488-labelled secondary antibody (ThermoFisher Scientific A-11018) and Dapi. Cells were mounted with Dako Fluorescent Mounting Medium (S3023). Images were captured with a LSM 880 confocal microscope (Zeiss).

Immunofluorescence staining was also performed on TA sections (12 µm) after fixation with 4% paraformaldehyde and blocking with 5% horse serum in PBS. Sections were incubated with a laminin (1:250, Santa Cruz SC59854) primary antibody followed by Alexa Fluor 488-labelled secondary antibody (ThermoFisher Scientific A21208). Sections were mounted with Dako Fluorescent mounting medium (Dako S3023). Images were captured with an Axioscan (Zeiss).

### Immunostaining Analysis

The area of muscle fibers and the diameter of primary and C2C12 myofibers were quantified using a FiJi macro^43^ for image input and preprocessing functions. For the diameter of primary and C2C12 myofibers, between 60 and 160 fibers were analyzed for each condition, and 3 measures were taken manually for each fiber and averaged.

The Nuclear intensity of FoxO1 and FoxO3a staining was performed using Acapella^TM^ image analysis software (version 2.7-Perkin Elmer Technologies, Waltham, USA) by using dedicated module to detect nuclei shape in Dapi following by the intensity tracking by nucleus in channel FITC. A minimum of 100 cells per condition were analyzed in two independent experiments.

### Gene Expression Analysis

Total RNA was extracted using the guanidium thiocyanate/phenol/chloroform extraction method for frozen muscles and using trizol (15596018, Life technologies) for C2C12 cells as previously described^31^. DNAse treatment was performed (EN0521, Thermo Scientific) and cDNA was obtained using a reverse transcription kit (High-capacity cDNA reverse transcription Kit, Life Technologies). Quantitative qPCR was performed using specific primers (Supplemental Table 1) and the Brillant II SYBR Green QPCR Master Mix (Agilent Technologies). Results were analyzed with the standard delta Cycle Threshold method and normalized to the expression of *PPia* (*Cyclophilin A*).

### Chromatin ImmunoPrecipitation (ChIP)

ChIP experiments were performed as previously described^44^ using the anti-Rev-erb-α (1:50, #13418, Cell Signalling) and normal rabbit IgG (1:250, #2729, Cell Signalling) antibodies following manufacturer’s instructions. Briefly, differentiated C2C12 cells were crosslinked with 1% paraformaldehyde for 10 min. Extracted nuclei were resuspended in lysis buffer (Tris-HCl 50 mM pH8.0, EDTA 10 mM, SDS 1%, protease inhibitor cocktail). Chromatin was then sheared for 20 min using a water-cooled Bioruptor (Diagenode) and diluted 10 times with the dilution buffer (Tris HCl 20 mM pH8.0, Triton X-100 1%, Glycerol 5%, EDTA 2 mM, NaCl 150 mM, protease inhibitor cocktail from Roche). 100 µg of DNA were used per IP and recovered chromatin was purified using QIAquick PCR clean up kit (Qiagen). Immunoprecipitated DNA was analysed by qPCR and normalized to input samples and negative DNA region.

### Proteasome activity

The 20S Proteasome Activity Assay kit (APT, Millipore, Billerica, MA, USA) was used to measure the proteasome activity according to the manufacturer’s instructions.

### Statistics

To compare two groups, data were analysed using the Mann-Whitney test or the two-tailed unpaired student’s *t*-test after being assessed for normality. One-way ANOVA with Bonferroni post-hoc analysis was used to compare more than 2 groups. Qualitative traits (i.e. area frequency distribution) were analysed with a Chi^2^ test. Statistical significance is shown on the graphs (*p < 0.05; **p < 0.01; ***p < 0.001; ****p<0.0001). Statistical methodologies used for each data set are indicated in the figure legends. Prism 6.0 (GraphPad Software Inc., USA) was used for statistical analysis.

## Electronic supplementary material

Supplemental material
