# Supplementary material for: Rev-erb-α regulates atrophy-related genes to control skeletal muscle mass
Source: Sci Rep. 2017 Oct 30;7:14383. doi: 10.1038/s41598-017-14596-2 (PMC5662766; doi:10.1038/s41598-017-14596-2)

Supplemental Data

Rev-erb-α regulates atrophy-related genes to control skeletal muscle mass

Alicia Mayeuf-Louchart[#1], Quentin Thorel[#1], Stéphane Delhaye[1], Justine Beauchamp[1], Christian Duhem[1], Anne Danckaert[2], Steve Lancel[1], Benoit Pourcet[1], Estelle Woldt[1], Alexis Boulinguiez[1], Lise Ferri[1], Mathilde Zecchin[1], Bart Staels[1], Yasmine Sebti[§1], Hélène Duez[§1*]

[1] Univ. Lille, Inserm, CHU Lille, Institut Pasteur de Lille, U1011 - EGID, F-59000 Lille, France

[2] Institut Pasteur, Imagopole - CITech, Paris, France

[#],[§] equally contributed to the work

*Correspondence to: Helene.duez@pasteur-lille.fr

1, rue du Professeur Calmette, BP245, 59019 Lille cedex

Short title: Regulation of skeletal muscle atrophy-related genes by Rev-erb-α

Keywords: Rev-erb-α; skeletal muscle atrophy; proteasomal degradation; FoxOs; dexamethasone

Supplemental Table 1: List of specific primers used for quantitative PCR gene expression analysis

Gene Name	Forward Primer	Reverse Primer
Cyclophilin A	GCATACGGGTCCTGGCATCTTGTCC	ATGGTGATCTTCTTGCTGGTCTTGC
Klf15	AAGCCCTTTGCCTGCACCTGG	CGTACTGCGCGGCTGCTTCG
Atrogin1	GGGGTCACCCTGCAGCTTTGC	GGGGAAAGTGAGACGGAGCAGC
Bcat2	CCCTCCTGGCGGACCCTTCAT	AGCTGGTGGTCTGGCCCGT
Foxo1	CATCCACTCGTAGATCTGCG	TCGTCGCGCCGCAACGCGTG
Foxo3a	CCGGACAAACGGCTCACTT	GGTTGTGCCGGATGGAGTT
Redd1	TCTCGAACTCCGGCCGCTGA	CCCAATCGCGCCTGGGACAG
Murf1	ATGGACCGGCACGGGGTGTA	GCACATCGGGTGGCTGCCTT
Ubc	CGCGCTGATCCCTCCG	CTGCATCGTCTCTCTCACGG
Bmal1	GGACTTCGCCTCTACCTGTTC	ACCCGTATTTCCCCGTTC
Rev-erbα (Nr1d1)	TGGCCTCAGGCTTCCACTATG	CCGTTGCTTCTCTCTCTTGGG

Supplemental Table 2: List of specific primers used for ChIP experiments

Gene Name	Forward Primer	Reverse Primer
Murf1 (-2.5kb)	CCA GCC ACT TGG GTT TTT AG	GCT GGA GAT ATG GGT GGG TA
Bcat2 (-2kb)	GGCCCTTAGGGGAGAAGAGT	GAGTGTCCAGAGCCTGTTCC
Bmal1	GGACTTCGCCTCTACCTGTTC	ACCCGTATTTCCCCGTTC

Supplemental Figure 1: The body weight of Rev-erb-α deficient mice is unchanged.

Body weight of *Rev-erbα*$^{+/+}$ and *Rev-erbα*$^{-/-}$ mice (n=13, 13). Results are expressed as means ± sem; non-significant (ns) by Mann-Whitney test.

Supplemental Figure 2: Rev-erb-α controls atrophy-related genes in skeletal muscle.

A, RT-qPCR analysis of *Rev-erbα* expression in gastrocnemius and tibialis anterior muscles from mice injected intra-muscularly with a Rev-erb-α expressing or a control AAV vector. B, RT-qPCR analysis of atrophy-related gene expression in tibialis anterior muscle from mice injected intra-muscularly with a Rev-erb-α expressing or a control AAV vector (n=7 per group). Results are expressed as means ± sem; ** $P < 0.01$, *** $P < 0.001$ by Mann-Whitney test.

Supplemental Figure 3: Proteasomal activity is modulated by Rev-erb-α in skeletal muscle

A, Proteasomal activity in myogenic cells isolated from *Rev-erbα*$^{+/+}$ and *Rev-erbα*$^{-/-}$ mice (n=3) and, B, in C2C12 cells over-expressing Rev-erbα (*p*REV-ERB-α) and control cells (*pBabe*) (n=4,5). Results are expressed as means ± sem;* $P < 0.05$, by Mann-Whitney test.

Supplemental Figure 4: Rev-erb-α expression is induced upon dexamethasone treatment in skeletal muscle.

RT-qPCR analysis of *Rev-erbα* expression in skeletal muscle from mice treated with dexamethasone for 5 days (n=6 per group). Results are expressed as means ± sem; * *P* < 0.05, by Mann-Whitney test.

Supplemental Figure 5: Reduced skeletal muscle *Bmal1* expression upon pharmacological Rev-erb-α activation. RT-qPCR analysis of *Bmal1* gene expression in quadriceps muscle (n=5 per group) from mice treated with SR9009 (100 mpk) twice daily or vehicle for 3 days. Results are expressed as means ± sem;* *P* < 0.05, by Mann-Whitney test.

Figure S1

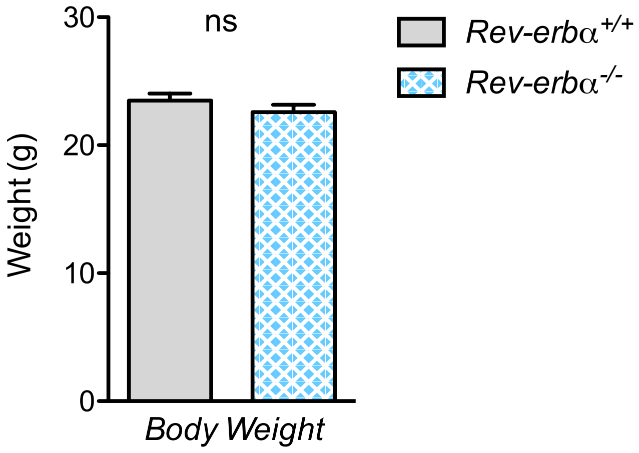

Figure S2

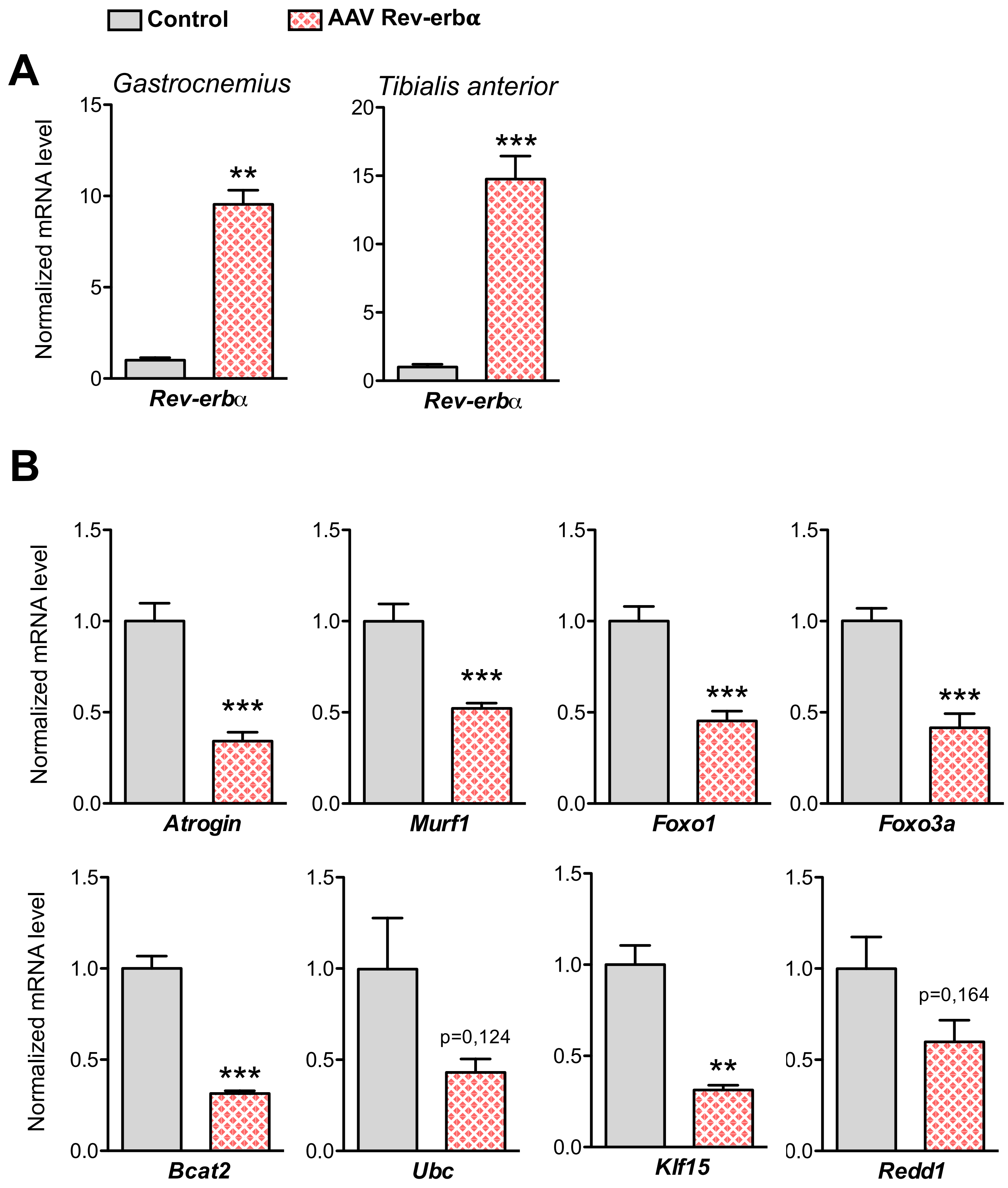

Control
AAV Rev-erbα
A
Gastrocnemius
Tibialis anterior
Normalized mRNA level
15
10
5
0
**
Rev-erbα
20
15
10
5
0
***
Rev-erbα
B
Normalized mRNA level
1.5
1.0
0.5
0.0
***
Atrogin
Murf1
Foxo1
Foxo3a
Bcat2
Ubc
p=0,124
Klf15
**
Redd1
p=0,164

Figure S3

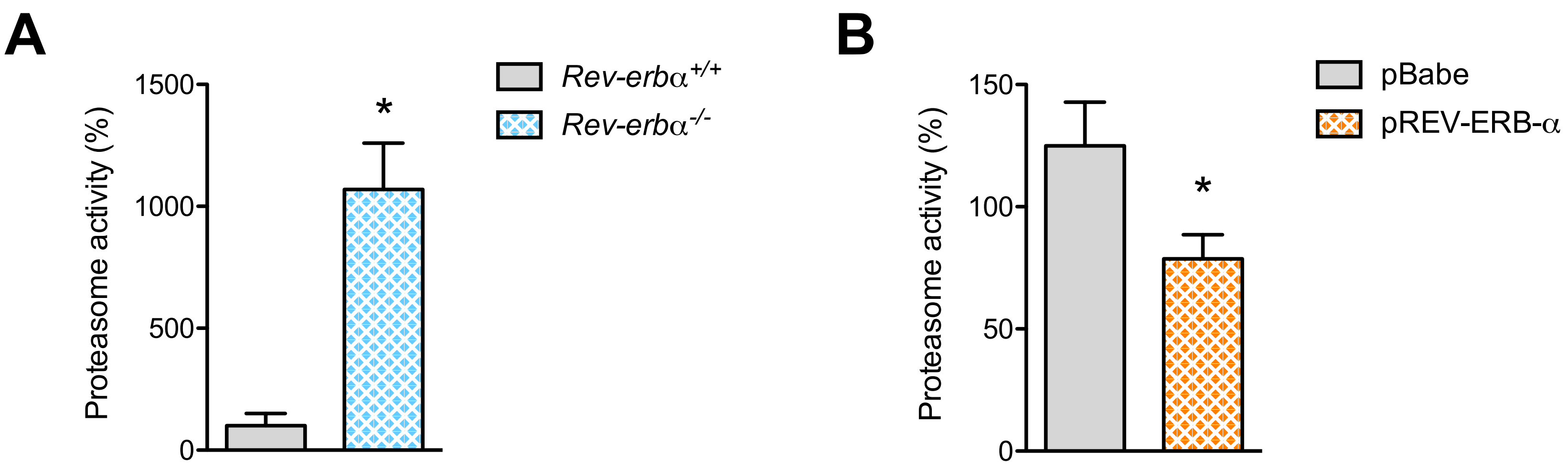

A
Proteasome activity (%)
1500
1000
500
0
*
Rev-erbα+/+
Rev-erbα-/-
B
Proteasome activity (%)
150
100
50
0
*
pBabe
pREV-ERB-α

Figure S4

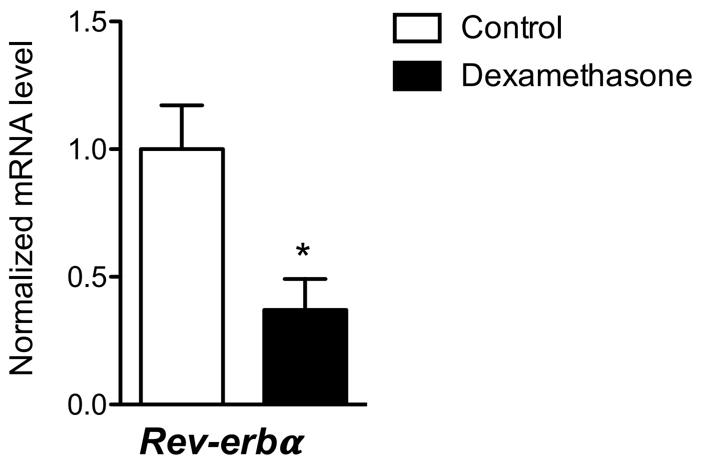

Normalized mRNA level
1.5
1.0
0.5
0.0
*
Rev-erbα
Control
Dexamethasone

Figure S5

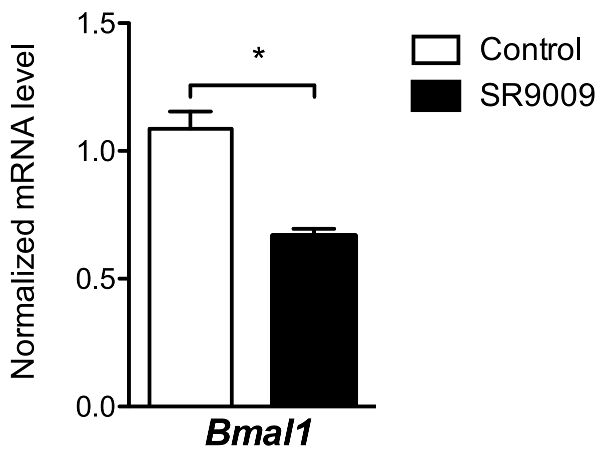

Supplement: Supplementary file 1 — Supplemental material [file 41598_2017_14596_MOESM1_ESM.pdf]
